# Investigation of Photorecoordination Kinetics for Complexes of Bis(aza-18-crown-6)-Containing Dienones with Alkali and Alkaline-Earth Metal Cations via Time-Resolved Absorption Spectroscopy: Structure vs. Properties

**DOI:** 10.3390/molecules30194005

**Published:** 2025-10-07

**Authors:** Oleg A. Alatortsev, Valeriy V. Volchkov, Mikhail N. Khimich, Ivan D. Sorokin, Mikhail Ya. Melnikov, Fedor E. Gostev, Ivan V. Shelaev, Victor A. Nadtochenko, Marina V. Fomina, Sergey P. Gromov

**Affiliations:** 1 Department of Chemistry, M. V. Lomonosov Moscow State University, 119991 Moscow, Russia; volchkov_vv@mail.ru (V.V.V.); khimichmn@my.msu.ru (M.N.K.); sorokinid@my.msu.ru (I.D.S.); melnikov46@mail.ru (M.Y.M.); 2 N. N. Semenov Federal Research Center for Chemical Physics, Russian Academy of Sciences, 119991 Moscow, Russia; fedor.gostev@chph.ras.ru (F.E.G.); ivan.shelaev@chph.ras.ru (I.V.S.); nadtochenko@gmail.com (V.A.N.); 3 Photochemistry Center, Kurchatov Complex of Crystallography and Photonics, National Research Center “Kurchatov Institute”, 119421 Moscow, Russia; mv_fomina@mail.ru

**Keywords:** bis(aza-18-crown-6)-containing dienone, complexation, photoactive supramolecular systems, fluorescence, photorecoordination, time-resolved absorption spectroscopy, TD DFT calculations

## Abstract

The analysis of time-resolved S_1_–S_n_ absorption spectra in the 0–500 ps range, together with quantum-chemical calculations, uncovered a photorecoordination reaction for the following complexes of CD6 (a bis(aza-18-crown-6)-containing dienone (ketocyanine dye) with a central cyclohexanone fragment): CD6·(M^n+^)_2_ (M = Ba^2+^, Sr^2+^, Ca^2+^, K^+^). This process takes place over hundreds of fs and involves an “axial-to-equatorial” conformational change, with the solvation shell undergoing rearrangement as well. The characteristic photorecoordination times were found to correlate with the stability constants of the complexes. The lifetimes for the fluorescent states of CD6 and its complexes, namely CD6·(M^n+^)_2_ (M = Ba^2+^, Sr^2+^, Ca^2+^, K^+^), are different; ergo, there is no photoejection of crowned cations into the solution. The calculated conformational profiles in the ground and excited states indicate the presence of an energy barrier in this process. A general photorelaxation pathway is suggested for CD6·(M^n+^)_2_ metal complexes (M = Ba^2+^, Sr^2+^, Ca^2+^, K^+^). The coordination of cations via the carbonyl moiety in the dye molecule promotes photorecoordination of metal cations in the cavities of the azacrown ether fragment. Photorecoordination times were found to correlate with the degree of conjugation between the lone pairs in the N atoms of the aza-18-crown-6 ether and the π subsystem in the dye molecules (established for the CD4–CD6 metal–dye complex series, where CD4 and CD5 are related dyes with central cyclobutanone and cyclopentanone fragments, respectively).

## 1. Introduction

**Scheme 1:**
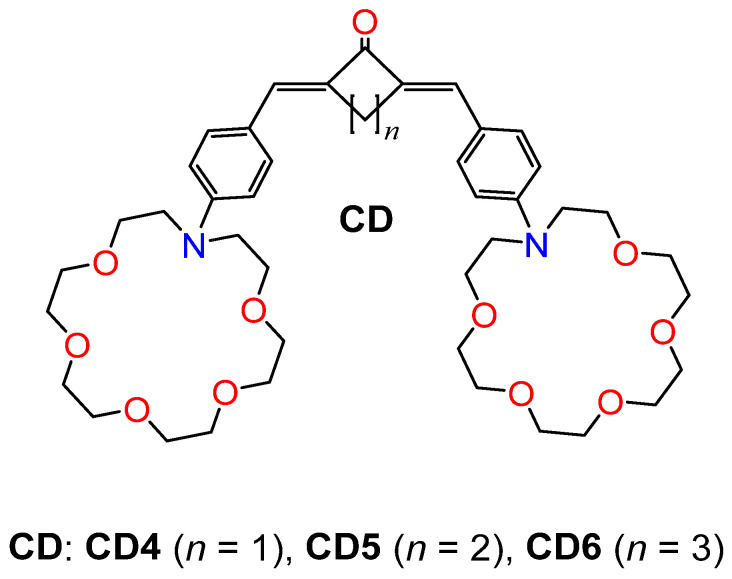
Structure of CD4–CD6.

Ketocyanine dyes are of great interest to researchers due to the extent of their application in biology, medicine, and the designing of photoactive materials [1,2,3,4,5,6]. Previously, our research group reported on the spectral-luminescent behavior of the CD6 ketocyanine dye (*n* = 3) (Scheme 1) and three types of its complexes with alkali and alkaline-earth metal cations: (CD6·(M^n+^), CD6·(M^n+^)_2_, and M^n+^·CD6·(M^n+^)_2_) [7].

**Scheme 2:**
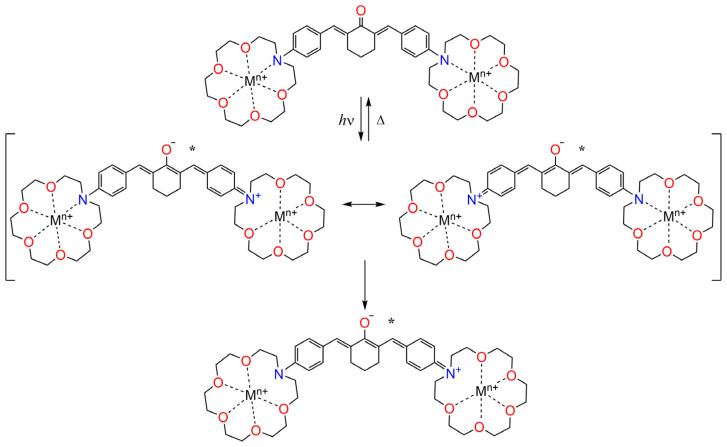
CD6 dye in its ground and excited (*) states.

According to our data, complexes with high stability constant (lg*K*_s_^1:2^ > 2.5)-CD6·(M^n+^)_2_ (M^n+^ = Ba^2+^, Sr^2+^, Ca^2+^, K^+^) undergo photoinduced recoordination of the metal cations in the azacrown ether cavities, wherein the N⋯M^n+^ bonds dissociate (Scheme 2). A similar process takes place for CD4·(M^n+^)_2_, CD5·(M^n+^)_2_ complexes (M^n+^ = Ba^2+^, Ca^2+^, K^+^) [7,8]. As demonstrated previously, time-resolved absorption spectroscopy can help uncover the kinetic parameters of these processes, whereas quantum-chemical calculations (DFT and TD-DFT) provide evaluations for the prospective conformational changes during photorecoordination.

**Scheme 3:**
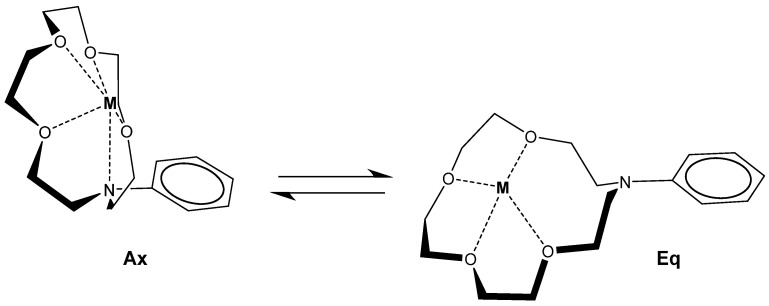
Conformational changes in the molecule during photorecoordination: from “axial (Ax)” to “equatorial (Eq)” forms.

In general, earlier reports were concerned with aza (15-crown-5) ether-based dyes. Supposedly, for these compounds, photorecoordination reaches the elimination stage, wherein the weakly bonded cation is ejected into the solution. Thus, for a complex involving aza (15-crown-5)-containing merocyanine, Ca^2+^ photoejection was observed in the ps range [9]. At ~130 fs, upon the dissociation of the N⋯Ca^2+^ bond, a so-called “loose complex” was produced. The presence of a stimulated emission (SE) band for the complex undergoing a bathochromic shift with the characteristic time of ~670 ps was suggested as evidence of photoejection. Similarly, photoejection was discovered for the related Sr^2+^-based complex [10]. In [(2,2′-dipyridyl)Re(CO)_3_L]^+^ complexes with Li^+^, Na^+^, Ca^2+^, and Ba^2+^ cations (notably, L contains an aza (15-crown-5) ether fragment), the L-Re charge transfer (metal–ligand charge transfer) resulted in photoejection on the ns scale [11]. The reverse process in the ground state proceeded in the μm scale. An analogous Mg^2+^ complex did not undergo photoejection, which was supported by the earlier TD-DFT calculations [12]. For the complex formed by 4′-monoaza-15-crown-5-flavonol with the Na^+^ cation, a state induced by intramolecular charge transfer (ICT) was rapidly vanished, stimulating photoejection of the cation. The authors detected ejection in complexes with varying stoichiometries: 1:1 (wherein the Na^+^ cation is coordinated via the aza (15-crown-5) ether fragment) and 1:2 (where the Na^+^ ion was additionally coordinated via the acceptor carbonyl moiety in the dye molecule). In both cases, photoejection was observed on the ps scale (<10 ps) [13]. In the ns range, Ba^2+^ underwent photoejection in the complexes of trans- and cis-isomers of a benzothiazole-type styryl dye that contained an aza (15-crown-5) ether fragment [14,15]. It was hypothesized that the molecule underwent conformational changes during photorecoordination, which was supported by quantum-chemical calculations (Scheme 3) [7,8,16].

Using bis(aza-18-crown-6)-containing dienone derivatives seems to be preferable to their bis(aza-15-crown-5)-containing analogs, since the cavity in the hexadentate macroheterocyclic ligand is larger than that in the pentadentate one. Therefore, this promotes the formation of significantly more stable complexes with a wider range of metal cations (Ba^2+^, Sr^2+^, Ca^2+^, K^+^, Mg^2+^, Na^+^, and Li^+^). Furthermore, this provides additional hindrances for photoejection of metal cations into the solvent during reversible photorecoordination, making the complexes of bis(aza-18-crown-6)-containing dienones prone to more effective supramolecular photoswitching. This could prove useful for producing photoactive supramolecular devices [17,18,19,20,21]. This present study continues the investigation of photorecoordination in the metal complexes of CD4–CD6 dyes with alkali and alkaline-earth metal cations. Studying the evolution of singlet excited states for these complexes allowed us to deduce the characteristic times of those processes and suggest a general scheme for their photorelaxation. The results of TD-DFT calculations support our conclusions.

## 2. Results and Discussion

### 2.1. Time-Resolved Spectroscopy

#### 2.1.1. CD6 Dye

**Figure 1:**
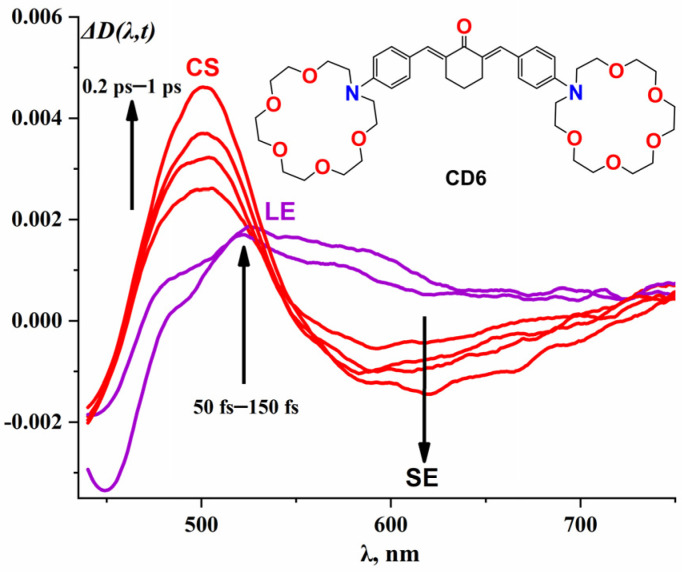
TA spectra for the CD6 dye in MeCN, 0–1 ps range, *λ*_exc_ = 430 nm.

The evolution of transient absorption (TA) spectra of the CD6 dye in MeCN involves the initial emergence of an intense broad band, followed by its narrowing and a hypsochromic shift of ~20 nm over 150 fs (Figure 1). Up until 1 ps, the intensity of the narrowed band keeps rising at 500 nm. After quenching in the ~2–500 ps range, monoexponential decay takes place, with the position and shape of the band remaining the same. The negative SE band at 595 nm appears in ~1 ps. Subsequently, a bathochromic shift of 35 nm carries on up to 4 ps, followed by complete decay by 500 ps. Presumably, the emergence of the band at 500 nm reflects the LE (local exited) ⟶ CS (charge shifted) reaction producing a state with partial negative charge transfer to the central fragment of CD6. The bathochromic shift of the SE band is consistent with the relaxation of the solvent around the CD6 molecule in its more polar CS state. The time plots of accumulation and decay for the TA bands corresponding to CD6 in MeCN in the 0–500 ps range, together with their monoexponential fitting, can be found in the Supplementary Materials (Figure S1).

#### 2.1.2. CD6·(H^+^)_2_ Complex

**Figure 2:**
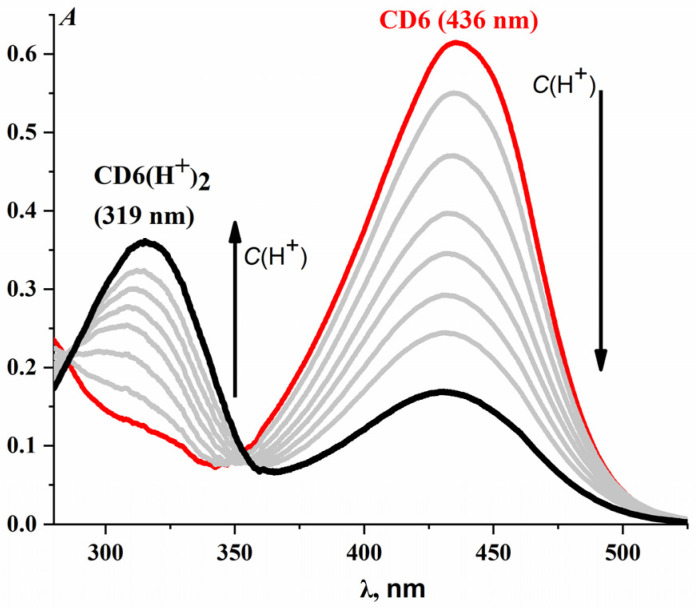
Absorption spectra for CD6·(H^+^)_2_ in MeCN (C(CF_3_COOH) from 0 to 10^−3^ M). Gray curves correspond for steadily increasing concentration of CF_3_COOH in solution, from 0 (CD6) to 10^−3^ M CD6·(H^+^)_2_.

In ~0.7 ps, a narrow TA band emerges for the CD6·(H^+^)_2_ complex at 408 nm. After a short quenching period (up to ~0.9 ps), it decays almost completely by 20 ps. An SE band could not be detected in the spectra. The chromophore of the CD6·(H^+^)_2_ complex is significantly shortened due to complete disintegration of π-to-π conjugation with N atoms in both azacrown ether fragments (the hypsochromic absorption shift upon the formation of CD6·(H^+^)_2_ amounts to 117 nm, Figure 2). This hinders charge transfer to the central fragment upon excitation. This may account for a significant hypsochromic shift of the TA maximum (92 nm) assigned to the LE band of the CD6·(H^+^)_2_ complex relative to the CS band of the unbound dye molecule. Photorecoordination does not take place in the protonated complex.

#### 2.1.3. CD6·(M^n+^)_2_ Complexes

The spectral evolution of highly thermodynamically stable CD6·(M^n+^)_2_ complexes is broadly similar to the one we just described. The key difference is in the characteristic TA band decay times. However, this process diverges from the evolution observed for the dye molecules (both in their free and protonated forms) since photorecoordination does not proceed in those cases.

This can be exemplified by the CD6·(Sr^2+^)_2_ complex. At first, a narrow TA band of the complex appears, with its maxima at 416 nm (intense peak) and ~550 nm (weak broadened peak, detectable only after 70 fs). The principal maximum at 416 nm keeps growing until 440 fs, subsequently decaying in ~15 ps. The low-intensity peak keeps growing until 130 fs (which resembles the characteristic time of the LE state for the dye). Conceivably, the band at 416 nm can be ascribed to the non-recoordinated state of the complex with the N⋯Sr^2+^ preserved, whereas the weak band at 550 nm can be ascribed to the short-lived LE state of the recoordinated complex with the N⋯Sr^2+^ bonds broken. The intense band at 500 nm corresponding to a fluorescent state of the CD6·(Sr^2+^)_2_ complex emerges at 0 to ~3 ps and, upon a period of quenching (up to ~9 ps), decays monoexponentially in the 9–500 ps range. Curiously, this band lies in the same spectral region as the CS band of the initial CD6 dye with their characteristic decay times evaluated at the same order of magnitude. This allows us to ascribe the band at 500 nm to the already recoordinated CS state of the CD6·(Sr^2+^)_2_ complex with the N⋯Sr^2+^ bonds destroyed and the chromophore in the dye molecule restored.

The weak and noisy negative SE band of the complex takes approximately 25 ps to emerge and decays completely in the 25–500 ps range. The nature of spectral evolution for CD6·(Sr^2+^)_2_ and CD6·(Ba^2+^)_2_ complexes is the same, pointing to identical influence that the Ba^2+^ and Sr^2+^ ions in the cavities of azacrown ether fragments have on the chromophore system of the dye molecule (the hypsochromic shifts in absorption upon the formation of CD6·(Sr^2+^)_2_ and CD6·(Ba^2+^)_2_ amounted to 90 and 91 nm, respectively). The evolution of TA spectra for the CD6·(Ca^2+^)_2_ complex involves the emergence of a band at 450 nm in ~350 fs, with its partial decay up until 4 ps. This can be ascribed to the non-recoordinated state. Afterwards, a band at 500 nm keeps rising until 2 ps with its intensity decreasing upon quenching by approximately 30% in the 25–500 ps range. This can be ascribed to the recoordinated state. The SE band at 600 nm emerges in 10 ps and decays completely in ~70–500 ps. The evolution of TA spectra for the CD6·(K^+^)_2_ complex resembles that for CD6. This supports the notion of the K^+^ cation having the lowest influence on the chromophore in the dye (as compared to Ba^2+^, Sr^2+^, and Ca^2+^), manifesting in a shift of 30 nm. In the 0–1.1 ps range, a band emerges at 495–500 nm with its complete decay in 1.1–500 ps. Additionally, an SE band arises at 600 nm in ~1 ps, with its subsequent decay in the 1–500 ps range and a bathochromic shift of 20 nm for the absorption maximum.

**Figure 3:**
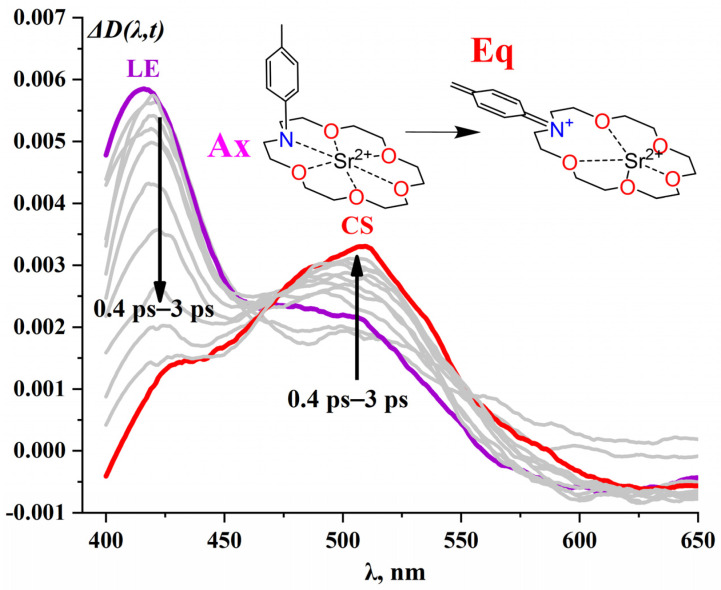
TA spectra for the CD6·(Sr^2+^)_2_ complex in the 0–3 ps range, *λ*_exc_ = 340 nm. Insert: “axial” (Ax)—“equatorial” (Eq) conformational change during photorecoordination. Gray curves correspond to changes across the 0.4–3 ps range.

As noted earlier [10], the bathochromic shift of the negative SE band over time can serve as an indicator of the distance and, correspondingly, the strength of the interaction between the metal cations and the N atoms in the azacrown ether fragments. Thus, such a shift manifesting in the ps range only for K^+^ (in the Ba^2+^, Sr^2+^, Ca^2+^, and K^+^ series) suggests that K^+^ cations are the furthest removed from the N atoms of the azacrown ether fragments during photorecoordination. This agrees with the lg*K*_s_^1:2^ = 2.72 values for CD6·(K^+^)_2_ being the lowest in the series. The fluorescence lifetimes for CD6 (0.167 ± 0.001 ns) and CD6(K^+^)_2_ (0.158 ± 0.002 ns) are close enough but not identical, confirming the initial suggestion. The spectral-kinetic characteristics of the TA spectra for CD6 and CD6·(M^n+^)_2_ (M^n+^ = Ba^2+^, Sr^2+^, Ca^2+^, K^+^) are given in Table 1, and the spectral evolution for the CD6·(Sr^2+^)_2_ complex can be seen in Figure 3. The time plots of onset and decay for the TA bands characterizing the CD6·(Sr^2+^)_2_ complex in MeCN in the 0–500 range and their monoexponential fittings are provided in the Supplementary Materials (Figure S2).

**Figure 4:**
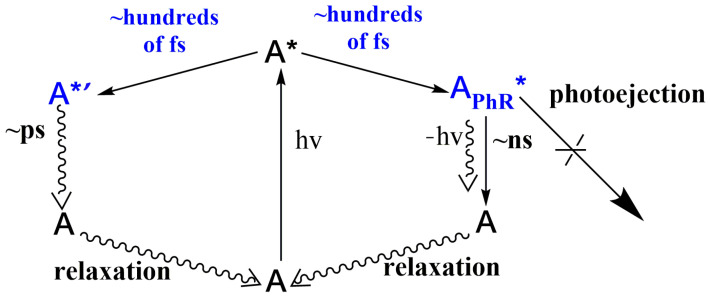
Relaxation pathways for excited CD6·(M^n+^)_2_ (M^n+^ = Ba^2+^, Sr^2+^, Ca^2+^, K^+^) complexes in MeCN. A*—initial hot LE state; A*’—non-recoordinated charge-shifted state; A_PhR_*—photorecoordinated state.

Table 1 clearly shows that 1:2-type complexes undergo photorecoordination in hundreds of fs with their characteristic times correlating with complex stability constants. Thus, in the Ba^2+^, Sr^2+^, Ca^2+^, and K^+^ series, the most stable CD6·(Ba^+^)_2_ complex takes the longest to recoordinate (934 fs), whereas the least stable CD6·(K^+^)_2_ complex is coordinated the fastest (181 fs). Fluorescence quantum yields of all complexes undergoing recoordination are a touch lower than those for the initial CD6 dye molecule, reflecting their less rigid molecular frameworks. The rate constants of non-radiative decay are many times higher than those for radiative decay; for example, this ratio is ~10 for the CD6·(Sr^2+^)_2_ complex. The lifetimes of fluorescent states for the dye and the complexes are different, indicating that cations are not ejected into the solution. Based on our current observations and calculations, we can conclude that, if lg*K*_s_^1:2^ > 1.8, photoejection of crowned cations into bulk solution does not elapse for CD4–CD6 complexes. The proposed relaxation pathway for the singlet excited states in metal complexes is provided in Figure 4, common for complexes of the CD6·(M^n+^)_2_ type (M^n+^ = Ba^2+^, Sr^2+^, Ca^2+^, K^+^).

#### 2.1.4. M^n+^·CD6·(M^n+^)_2_-Type Complexes

Generally, 1:3-type M^n+^·CD6·(M^n+^)_2_, (M^n+^ = Mg^2+^, Li^+^, Na^+^) complexes with additional cation coordination via the carbonyl group in the dye molecule exhibit different dynamics in the TA spectra in comparison to 1:2-type complexes with Ba^2+^, Sr^2+^, Ca^2+^, and K^+^. For example, the spectra of the Na^+^·CD6·(Na^+^)_2_ complex evolve as follows: in ~1.1 ps, a TA band emerges at 500 nm with subsequent quenching up until 5 ps and complete decay in the 5–500 ps range. A hypsochromic shift of 20 nm detected upon its onset indicates the decrease of conjugation in the chromophore of the dye molecule. The Li^+^·CD6·(Li^+^)_2_ system behaves similarly with the only differences being the positions of TA (535 nm) and SE (625 nm) maxima, as well as characteristic times of accumulation and decay for these bands. For the Mg^2+^·CD6·(Mg^2+^)_2_ complex, no TA bands could be detected. In the 2 ps range, an SE band emerges at 630 nm with its subsequent decay in the 2–500 ps range with the position of the maximum preserved. Spectral-kinetic parameters for the TA spectra of M^n+^·CD6·(M^n+^)_2_ (M^n+^ = Mg^2+^, Li^+^, Na^+^) complexes are given in Table 2.

**Figure 5:**
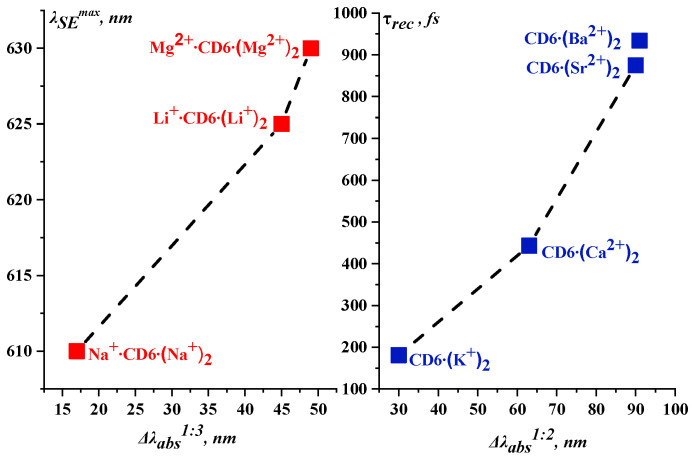
Location of SE band in the TA spectrum versus magnitude of bathochromic shift in the steady-state absorption spectra upon complexation for M^n+^·CD6·(M^n+^)_2_ (M^n+^ = Na^+^, Li^+^, Mg^2+^) (**left**). Photorecoordination time versus magnitude of hypsochromic shift in absorption spectra upon complexation for CD6·(M^n+^)_2_ (M^n+^ = Ba^2+^, Sr^2+^, Ca^2+^, K^+^) (**right**). *τ_rec_* corresponds to *τ*_1_ in Table 1.

Table 2 shows that M^n+^·CD6·(M^n+^)_2_, (M^n+^ = Mg^2+^, Li^+^, Na^+^) complexes exemplify correlation between the lifetime of a fluorescent state (*t*_3_), the position of an SE band, and the surface charge density of the cation. For Na^+^·CD6·(Na^+^)_2_ and Li^+^·CD6·(Li^+^)_2_, TA bands appear less rapidly than for CD6. The SE band for M^n+^·CD6·(M^n+^)_2_ (M^n+^ = Li^+^, Mg^2+^) has a more pronounced hypsochromic shift in comparison with CD6·(M^n+^)_2_ (M^n+^ = Ba^2+^, Sr^2+^, Ca^2+^, K^+^). This suggests a larger change in the distance between the cations and the N atoms during recoordination for the former complexes. Due to additional coordination of the cation via the carbonyl group, 1:3 complexes are more polarized. The disparities in excited-state lifetimes for the CD6 dye and the 1:3 complexes signify that, herein, cations are not photoejected into the solution. The relation between the location of the SE band in the spectrum and the magnitude of the bathochromic shift in the absorption spectra upon complexation for M^n+^·CD6·(M^n+^)_2_ (M^n+^ = Na^+^, Li^+^, Mg^2+^) can be seen in Figure 5. Additionally, Figure 5 depicts the correlation between photorecoordination times and the magnitude of hypsochromic shifts in absorption spectra upon complexation for CD6·(M^n+^)_2_ (M^n+^ = Ba^2+^, Sr^2+^, Ca^2+^, K^+^).

### 2.2. Quantum-Chemical Calculations

**Figure 6:**
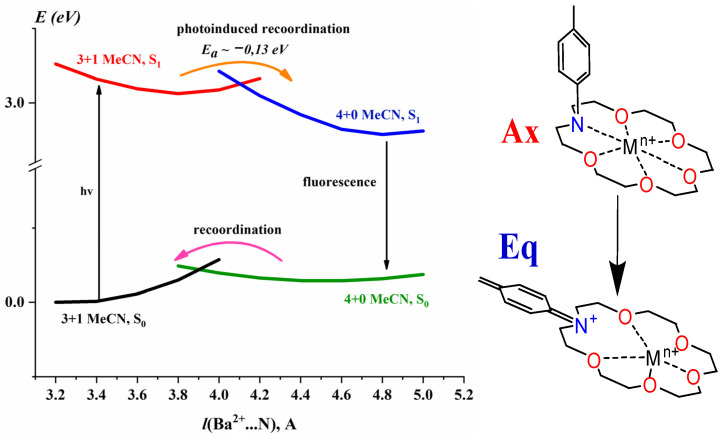
CD6·(Ba^2+^)_2_ energy as a function of Ba^2+^⋯N distance in ground and excited states (**left**); “axial (Ax)” to “equatorial (Eq)” conformational change or the complex during photorecoordination (**right**).

Since Ba^2+^–CD6 complexation is characterized by the largest hypsochromic shift of all detected (91 nm), this complex acted as a model object in our quantum-chemical calculations. According to the TD-DFT calculations, upon photorecoordination, the N⋯Ba^2+^ distance increases from 3.4 to 4.8 Å. The conformation of the metal complex changes from “axial” to “equatorial”, and the first solvation shell transforms as well: (3 + 1) MeCN to (4 + 0) MeCN. This process has an energy barrier both in its ground and excited states (*E*_a_~0.13 eV, Figure 6). The calculations demonstrated that binding the Mg^2+^ cation to the carbonyl group significantly lowered the energy of the photorecoordinated form of Mg^2+^·CD6·(Ba^2+^)_2_ (compared to the energy of the photorecoordinated form of CD6·(Ba^2+^)_2_), therefore allowing us to assume that it promoted the photorecoordination ability of Ba^2+^ crowned cations by means of Mg^2+^ coordination to the carbonyl group. Moreover, a small energy gap between the “axial” and “equatorial” conformations in the ground state of Mg^2+^·CD6·(Ba^2+^)_2_ implies that some amount of Mg^2+^·CD6·(Ba^2+^)_2_ species are already in “equatorial” (recoordinated) conformation in their ground state. Notably, we also demonstrated that using PrCN as a solvent at 77 K prohibits photorecoordination for CD5·(M^n+^)_2_ (M^n+^ = Ba^2+^,Ca^2+^, K^+^) [8], and the same is relevant for CD6·(M^n+^)_2_ (M^n+^ = Ba^2+^, Sr^2+^, Ca^2+^, K^+^) [7]. Consequently, we may assume that an on/off switch for photoinduced recoordination was discovered.

### 2.3. Structure vs. Properties for CD4–CD6 Dyes

As reported earlier [7], switching the central cyclohexanone fragment (CD6) with a cyclopentanone (CD5) or a cyclobutanone (CD4) fragment significantly affects the degree of conjugation between the lone pairs (LPs) in the N atoms of the aza-18-crown-6 ether fragments to the π subsystem in the dye molecule. This may affect the efficiency of complexation and photorecoordination. Indeed, the values of electron affinity (EA) for cyclobutanone (1 × 10^−3^ eV), cyclopentanone (1.69 × 10^−3^ eV), and cyclohexanone (3.3 × 10^−3^ eV) demonstrate that the former is the best electron acceptor, which, among other things, manifests in the values of complex stability constants [22]. The CD6·(M^n+^)_2_ complexes with Ba^2+^, Ca^2+^, and K^+^ ions are characterized by much higher stability constants than those for analogous CD4·(M^n+^)_2_ complexes. This is due to the LP in nitrogen atoms being more involved in π system conjugation for the CD4 dye molecule. This weakens the N⋯M^n+^ bonds in CD4·(M^n+^)_2_ to a larger extent compared with CD6·(M^n+^)_2_. Such behavior agrees with the *λ*_a_^max^ values for these dyes (Table 3): the higher the degree of conjugation in the molecule of the dye, the larger the bathochromic shift for the long-wave absorption band maximum. We were curious to compare the evolutions of TA spectra for these dyes. In the end, they were broadly similar, with the only differences manifesting in the values of spectral-kinetic parameters.

**Figure 7:**
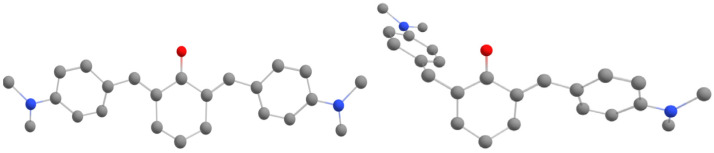
Planar (**left**) and twisted (**right**) model dye molecule conformations (CD6 with no azacrown ether fragments). C—gray dots, O—red dots, and N—blue dots; H atoms are omitted for clarity.

Table 3 shows that, in the CD4–CD6 series, fluorescence lifetimes and quantum yields decrease significantly, with non-radiative deactivation constants (*k*_d_) rising greatly. Quantum chemical calculations provide a possible explanation for that. In its twisted state, the dye molecule predominantly undergoes non-radiative decay. Twisting activation energy (*E*_a_) for CD6 is ~33% lower than that for CD4, with exoergicity of the process amounting to ~0.05 eV for CD4 versus 0.39 eV for CD6. Thus, the decrease in the degree of conjugation within the CD4–CD6 series promotes twisting for one of the fragments in the C=CH—Ar molecule, as depicted in Figure 7.

We were curious to compare the spectral-kinetic parameters of CD4·(M^n+^)_2_ and CD6·(M^n+^)_2_ metal complexes, with the former representing the highest degree of conjugation and the latter exemplifying the lowest (Table 4). This was necessary to investigate the influence that the degree of conjugation between the LP in the N atoms of the azacrown ether fragments to the π subsystem in the dye molecule may have on the photorecoordination process.

The data in Table 4, together with the corresponding values for CD6·(M^n+^)_2_ (M^n+^ = Ba^2+^, Ca^2+^, K^+^), suggest that, in the CD4–CD6 series, stability constants (lg*K*_s_^1:2^) and characteristic recoordination times (*t*_1_) increase, with fluorescent state lifetimes (*t*_2_) and fluorescence quantum yields (φ_f_) decreasing correspondingly. The rate constants for non-radiative deactivation achieve their maximum values for CD6·(M^n+^)_2_. In the CD4–CD6 series, the highest Stokes shifts are observed for CD6-containing metal complexes with Ba^2+^ and Ca^2+^ ions. This hints at more pronounced conformational changes in the excited states of relaxed forms of the CD6 complexes as compared to complexes formed by other dye molecules. Consequently, by varying the efficiency of conjugation between the LP in the N atoms of the aza-18-crown-6 ether fragments to the π subsystem in the dye molecule, we can affect characteristic photorecoordination times, switching between the radiative and non-radiative deactivation channels. The behavior for hybrid M^n+^·CD·(M^n+^)_2_ complexes in the CD4–CD6 series is broadly similar (see Table 5).

Additionally, Table 5 demonstrates that there is positive correlation between the strength of the Mg^2+^–carbonyl bond (lg*K*_s_^1:3^ decreases from CD4 to CD6) and the onset rate for the spectral band assigned to the recoordinated state. Consequently, we may assume that this bond promotes recoordination—which is also supported by model quantum-chemical calculations for the mixed Mg^2+^·CD6·(Ba^2+^)_2_ complex.

### 2.4. Thermally Induced Recoordination

**Figure 8:**
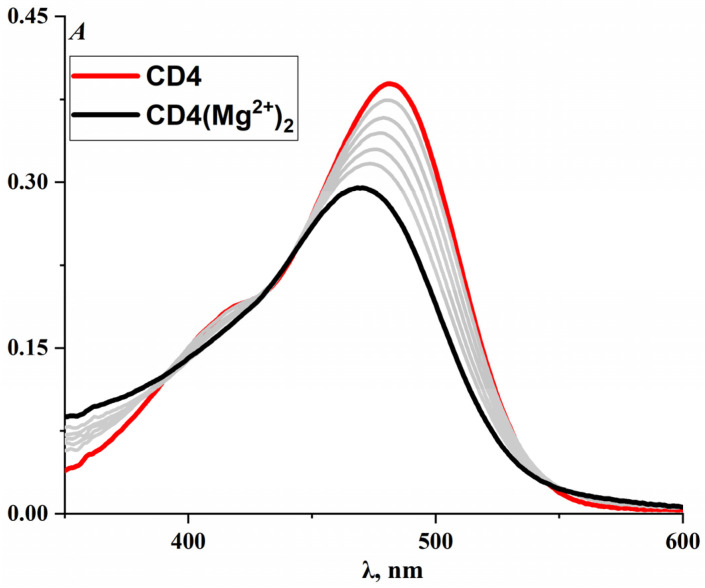
CD4 absorption spectra in the presence of Mg^2+^ cations in MeCN, *C* (Mg^2+^) in the 0 to 10^−3^ M range. Gray curves demonstrate a steady increase for Mg^2+^ concentration in the solution, from 0 (CD4) to 10^−3^ M (CD4·(Mg^2+^)_2_).

In photoactive crown ether-containing complexes, recoordination may also be initiated thermally, proceeding mostly for cations of smaller sizes. Earlier [23], such recoordination was observed for complexes formed by an *N*-methyl-aza-18-crown-6-containing styryl dye molecule with Li^+^ and Mg^2+^ ions. An additional band corresponding to a recoordinated state arose in the absorption spectra of those complexes. For CD6·(Li^+^)_2_, CD6·(Mg^2+^)_2_ and CD6·(Na^+^)_2_ complexes, thermally induced recoordination could not be found [7]. It was pertinent to check analogous CD4 complexes for such a possibility since the degree of conjugation between the LP in the N atoms of the azacrown ether fragments to the π subsystem in the dye molecule for these systems is significantly higher than that for CD6 [24]. Alas, CD4·(Li^+^)_2_, CD4·(Mg^2+^)_2_ and CD4·(Na^+^)_2_ complexes did not exhibit such behavior: only single bands could be detected in their absorption spectra, hypsochromically shifted in relation to the CD4 band (Figure 8).

## 3. Materials and Methods

### 3.1. Materials and Synthesis

The investigated bis(aza-18-crown-6)-containing ketocyanine dyes (dienones CD4, CD5 and CD6) were synthesized in accordance with known approaches [7,24]. Spectrally pure anhydrous Li, Na, K, Mg, Ca, Sr, and Ba perchlorates (Merck KGaA, Darmstadt, Germany) were used to produce supramolecular complexes. Heating in a VacuCell vacuum desiccator (MMM Medcenter, München, Germany) (0.01 mm Hg) at 200 °C for 5 h was used to remove water from the salts.

### 3.2. Time-Resolved Absorption Spectra

Freshly produced MeCN (“Panreac”, HPLC grade, water content 0.02 %*w*/*w*) solutions of dyes and their complexes were used in time-resolved spectroscopical experiments. Time-resolved S_1_ ⟶ S_n_ TA spectra were detected with a fs-resolution pulse differential absorption spectrometer at the N. N. Semenov Federal Research Center for Chemical Physics, Russian Academy of Sciences. The exact details of time-resolved spectroscopy measurements are given in [25]. For excitation, Gaussian pulses with ~60 GHz PRF, ~40 fs duration, and ~350 mJ energy were used (radiation maxima at 325, 340, 370, 417, and 430 nm). Pump and probe pulse polarizations were oriented at a “magic angle” (54.7°) against each other. The measured ∆*D* (λ,*t*) differential spectra were corrected accounting for continuum group velocity dispersion, following the procedure described in [25]. The experiments were carried out at 22 °C in a 0.5 mm optical cell. Fluorescence quantum yields were calculated via comparing the areas beneath the corrected fluorescence spectra of the samples in MeCN and the spectra of quinine bisulfate in 1 N H_2_SO_4_ (*φ*_f_ = 0.546) according to the following formula [26]:

*φ*_x_ = *φ*_f_ (*S*_x_/*S*_f_)((1 − 10^−*D*f^)/(1 − 10^−*D*x^)) (1)

where *φ*_f_ is the quinine bisulfate fluorescence quantum yield, *S_i_* represents the areas beneath the fluorescence spectra, and *D_i_* is the absorbance at excitation wavelength. The “x” and “f” indexes correspond to the investigated substance and quinine bisulfate, respectively.

### 3.3. TD-DFT Computations

Quantum-chemical calculations were carried out in Firefly 8.2 and Orca 5.0 packages [27,28]. For potential energy profiles, the geometries of CD6 and its complexes with M^n+^ were optimized in the ground electron state with a fixed N–M^n+^ distance via DFT (BHHLYP, 6-31G(d,p)) for all atoms except barium, for which the def2-SVP and def2-ECP levels were used [29,30]. The optimized geometric parameters for the ground and excited states of CD6 and its complexes were calculated via DFT and TD DFT with the BHHLYP functional and the same basis. Solvation was accounted for with the SMD model [31]. The energies of ground and excited states were also determined via DFT + SMD and TDDFT + SMD with the B3LYP functional and the same basis amended with diffuse functions for the nitrogen and oxygen atoms [32,33].

## 4. Conclusions

In CD6·(M^n+^)_2_ (M^n+^ = Ba^2+^, Ca^2+^, Sr^2+^, K^+^) complexes, photorecoordination proceeds in hundreds of fs, competing with non-radiative deactivation of non-recoordinated states. Characteristic photorecoordination lifetimes correlate with stability constants for these complexes. In those processes, “axial” complex conformations transform into “equatorial” ones, while the solvation shell changes from (3 + 1) MeCN to (4 + 0) MeCN. In the ground and excited states, recoordination proceeds via energy barriers. Photorecoordination is promoted via efficient bonding between the metal cation and the carbonyl moiety in the dye molecule. Cations do not undergo photoejection into the solution. A general photorelaxation pathway is suggested for CD6·(M^n+^)_2_ (M^n+^ = Ba^2+^, Ca^2+^, Sr^2+^, K^+^) metal complexes. For M^n+^·CD6·(M^n+^)_2_ (M = Mg^2+^, Li^+^, Na^+^) complexes, the position of the SE band and its lifetime is found to correlate with the magnitude of the bathochromic shift in absorption spectra upon complexation. In the CD4–CD6 series of dye molecules, a correlation is established between characteristic photorecoordination time and degree of conjugation between the LP in the N atoms of azacrown ether fragments and the π subsystem in the dye molecule. These results show the possibility of employing bis(aza-18-crown-6)-containing dibenzylidene cycloalkanones as prototype photoswitchable supramolecular devices.

## Figures and Tables

**Table 1:** TA (*λ*_TA_^max^) and SE (*λ*_SE_^max^) maxima for the bands corresponding to the CD6 dye molecule and the CD6·(M^n+^)_2_ (M^n+^ = Ba^2+^, Sr^2+^, Ca^2+^, K^+^) complexes. Characteristic times of accumulation (*t*_1_) and decay (*t*_2_, *t*_3_) for the TA bands, stability constants for type 1:2 complexes (lg*K*_s_^1:2^) ^b^ in MeCN at 22 °C.

System	*λ*_TA_^max^, nm	*t*_1_, fs	*t*_2_, ps	*t*_3_, ns	lg*K*_s_^1:2 b^	*λ*_SE_^max^, nm	*φ*_f_ ^c^	*k*_f_ × 10^−8^, c^−1^	*k*_d_ × 10^−9^, c^−1^
CD6	500	236 ± 4	—	0.167 ± 0.001	—	630	~0.03	1.79	5.79
CD6·(Sr^2+^)_2_ ^a^	416	124 ± 3	2.71 ± 0.07	—	5.29	~600	~0.01	0.80	7.92
	500	875 ± 25	—	0.125 ± 0.004					
CD6·(Ba^2+^)_2_	417	111 ± 2	2.94 ± 0.07	—	5.57	~600	<0.01	— ^d^	— ^d^
	500	934 ± 97 ^e^	27 ± 7	—					
CD6·(Ca^2+^)_2_	450	102 ± 6 ^f^	1 ± 0.1 ^f^	—	4.29	~600	~0.02	0.41	1.74
	500	443 ± 10 ^f^	—	0.562 ± 0.029					
CD6·(K^+^)_2_	— ^g^	—	—	—	2.72	~620	~0.02	1.20	6.20
	495	181 ± 4	—	0.158 ± 0.002					
CD6·(H^+^)_2_	408	191 ± 5	2.21 ± 0.05	— ^d^	— ^d^	— ^d^	— ^d^	— ^d^	— ^d^

^a^ The concentrations of metal perchlorates (M^n+^ = Ba^2+^, Sr^2+^, Ca^2+^, K^+^) are such that the concentration ratio CD6·(M^n+^)_2_/CD6·M^n+^ was ~94:1. ^b^ The corresponding values of stability constants for metal complexes (lg*K*_s_^1:2^) were published earlier [7]. ^c^ The concentrations of salts used in the calculations of fluorescence quantum yields are the following: ~10^−3^ M (Ba(ClO_4_)_2_), ~10^−3^ M (Sr(ClO_4_)_2_), ~5 × 10^−3^ M (Ca(ClO_4_)_2_), and ~10^−2^ M (KClO_4_). When recording the TA spectra, the excitation wavelengths were the following: 430 nm (CD6), 340 nm (CD6·(Ba^2+^)_2_ and CD6·(Sr^2+^)_2_), 370 nm (CD6·(Ca^2+^)_2_), 417 nm (CD6·(K^+^)_2_), and 325 nm (CD6·(H^+^)_2_). ^d^ “—” denotes that the parameter could not be deduced due to specific behavior in the system or is absent for this system. ^e^ Calculated from the time plot for Δ*D* (500 nm, *t*). ^f^ Calculated from the time plot for Δ*D* (450 nm, *t*), Δ*D* (500 nm, *t*). This is due to the overlap with background dynamics in the initial range, increasing the S (area) value: the calculation is incorrect due to overlap with the SE band. ^g^ The maximum for the first absorption band in the complex is the same as the wavelength of excitation.

**Table 2:** Spectral-kinetic parameters for the TA spectra of M^n+^·CD6·(M^n+^)_2_ (M^n+^ = Mg^2+^, Li^+^, Na^+^) complexes.

System	*λ*_exc_, nm	*λ*_TA_^max^, nm	*t*_1_, fs	*t*_3_, ns	lg*K*_s_^1:3 a^	*λ*_SE_, nm	q/d, 1/Å ^b^
Na^+^·CD6·(Na^+^)_2_	440	- ^c^	-	-	~0	610	~0.14
		500	454 ± 8	0.217 ± 0.002			
Li^+^·CD6·(Li^+^)_2_	515	- ^c^	-	-	~0	625	~0.58
		535	500 ± 5	0.153 ± 0.001			
Mg^2+^·CD6·(Mg^2+^)_2_	515	- ^c^	-	-	~0.32	~630	~0.91
		-	191 ± 7	0.088 ± 0.001 ^d^			

^a^ The corresponding values of stability constants for metal complexes (lg*K*_s_^1:3^) were published earlier [7]. ^b^ Surface charge density in the cation calculated as a ratio of its charge to its diameter. ^c^ Upon excitation of 1:3-type complexes, no TA band could be detected. ^d^ Calculated from the time plot of SE band decay at 620 nm (using areas).

**Table 3:** Spectral-kinetic parameters for CD4–CD6 in MeCN at 22 °C.

System	*λ*_a_^max^, nm	*λ*_TA_^max^, nm	*λ*_SE_^max^, nm	*t*_1_, fs	*t*_3_, ns	*φ*_f_ ^a^	*k*_f_ × 10^−8^, s^−1^	*k*_d_ × 10^−9^, s^−1^
CD4	475	523	596	~430	1.33	~0.20	1.50	0.60
CD5	466	511	605	~340	0.77	~0.13	1.70	1.13
CD6	436	500	630	~236	0.17	~0.03	1.79	5.79

^a^ Data related to CD4 and CD5 were reported earlier [5,6]. *t*_2_ (ps time scale) is omitted because these parameters are only relevant for the metal complexes of CD4–CD6.

**Table 4:** Spectral-kinetic parameters of CD4·(M^n+^)_2_ (M^n+^ = Ba^2+^, Ca^2+^, K^+^).

System	lg*K*_s_^1:2^	∆*λ*_a_^1;2^, nm	*λ*_f_^max^, nm	*λ*_TA_^max^, nm	*λ*_SE_^max^, nm	*t*_1_, fs	*t*_3_, ns	*φ*_f_	*k*_f_ × 10^−8^, s^−1^	*k*_d_ × 10^−9^, s^−1^
CD4·(Ba^2+^)_2_	3.68	104	612	504	~598	~520	~1.55	0.169	1.09	0.50
CD4·(Ca^2+^)_2_	4.05	33	604	496	~598	~230	~1.53	0.151	0.99	0.56
CD4·(K^+^)_2_	1.77	30	609	~502	~605	~250	~0.98	0.085	0.87	0.93

Data in Table 4 were reported earlier [8].

**Table 5:** Spectral-kinetic parameters for M^n+^·CD4·(M^n+^)_2_-, M^n+^·CD5·(M^n+^)_2_-, and M^n+^·CD6·(M^n+^)_2_-type complexes.

System	∆*λ*_a_^1:3^, nm	*λ*_TA_^max^, nm	*t*_1_, fs	*t*_3_, ns	lg*K*_s_^1:3^	*λ*_SE_^max^, nm
Mg^2+^·CD4·(Mg^2+^)_2_	−52	497	~130	0.82	2.1	644
Mg^2+^·CD5·(Mg^2+^)_2_	−59	490	~140	0.47	1.9	607
Mg^2+^·CD6·(Mg^2+^)_2_	−49	–	191 ± 7	0.088	~0.3	630

## Data Availability

The time-dependent absorption spectroscopy files are available from the respective authors upon reasonable request.

## Supplementary Materials

The following supporting information can be downloaded at https://www.mdpi.com/article/10.3390/molecules30194005/s1, Figure S1: TA band (CD6 in MeCN) accumulation at 500 nm, time plot, and its monoexponential fitting: y = 0.234 × exp (−*t*/0.236), where S*_i_* is the area beneath the TA band, 460–550 nm (a). TA band (CD6 in MeCN) decay at 500 nm, time plot, and its monoexponential fitting: y = 0.233 × exp (−*t*/167), where S*_i_* is the area beneath the TA band, 460–550 nm (b). *λ*_exc_ = 430 nm, T = 295 K; Figure S2: TA band (CD6∙(Sr^2+^)_2_ in MeCN) accumulation at 416 nm, time plot, and its biexponential fitting: y = −0.344 × exp (−*t*/0.124) + 0.26 × exp (−*t*/2.71) + 0.026, where S*_i_* is the area beneath the TA band, 400–450 nm (a). TA band (CD6·(Sr^2+^)_2_ in MeCN) accumulation at 500 nm, time plot, and its monoexponential fitting: y = 0.112 × exp (−*t*/0.87) + 0.006, where S*_i_* is the area beneath the TA band, 500–650 nm (b). TA band (CD6·(Sr^2+^)_2_ in MeCN) decay at 500 nm, time plot, and its monoexponential fitting: y = 0.156 × exp (−*t*/125) + 0.095, where S*_i_* is the area beneath the TA band, 400–650 nm (c). *λ*_exc_ = 340 nm, T = 295 K; Figure S3: Partial CD6·(Ba^2+^)_2_ structure: prior to photoinduced recoordination (axial conformation, (3 + 1) MeCN solvation shell) (left) and after photoinduced recoordination (equatorial conformation, (4 + 0) MeCN solvation shell) (right). Ba^2+^ (green), N (blue), O (red), and C (grey); H atoms are omitted for clarity.
