# Supplementary material for: Investigation of Photorecoordination Kinetics for Complexes of Bis(aza-18-crown-6)-Containing Dienones with Alkali and Alkaline-Earth Metal Cations via Time-Resolved Absorption Spectroscopy: Structure vs. Properties
Source: Molecules. 2025 Oct 7;30(19):4005. doi: 10.3390/molecules30194005 (PMC12525959; doi:10.3390/molecules30194005)
Supplement: Supplementary file 1 [file molecules-30-04005-s001.zip › molecules-3824087-supplementary.pdf]

Supporting Information for Publication

Investigation of Photorecoordination Kinetics for Complexes of Bis(aza-18-crown-6)-Containing Dienones with Alkali and Alkaline-Earth Metal Cations via Time-Resolved Absorption Spectroscopy: Structure vs. Properties

Oleg A. Alatortsev [1,*], Valeriy V. Volchkov [1], Mikhail N. Khimich [1], Ivan D. Sorokin [1], Mikhail Ya. Melnikov [1], Fedor E. Gostev [2], Ivan V. Shelaev [2], Victor A. Nadtochenko [2], Marina V. Fomina [3] and Sergey P. Gromov [1,3,*]

[1] Department of Chemistry, M. V. Lomonosov Moscow State University, 119991 Moscow, Russia; oleg.alatortsev@chemistry.msu.ru (O.A.A.); spgromov@mail.ru (S.P.G.)

[2] N. N. Semenov Federal Research Center for Chemical Physics, Russian Academy of Sciences, 119991 Moscow, Russia

[3] Photochemistry Center, Kurchatov Complex of Crystallography and Photonics, National Research Center "Kurchatov Institute", 119421 Moscow, Russia

* Correspondence: oleg.alatortsev@chemistry.msu.ru (O.A.A.); spgromov@mail.ru (S.P.G.)

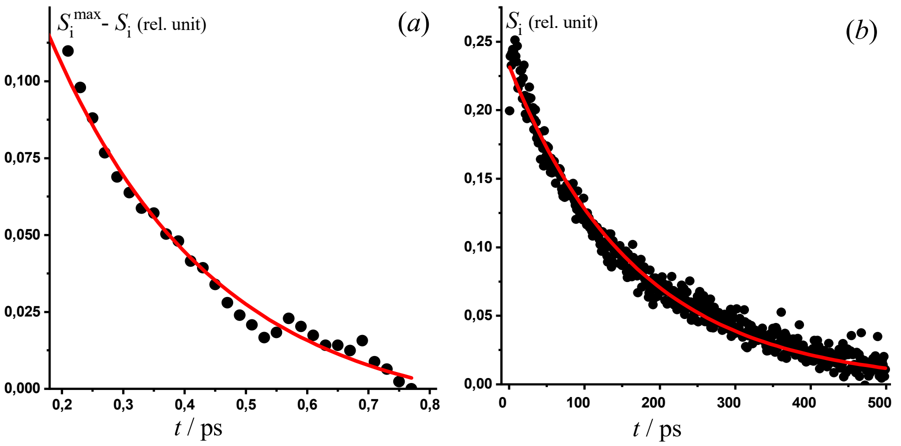

Figure S1. TA band (**CD6** in MeCN) accumulation at 500 nm, time plot and its monoexponential fitting: y = 0.234×exp(–*t*/0.236), where S_i is the area beneath the TA band, 460–550 nm (*a*). TA band (**CD6** in MeCN) decay at 500 nm, time plot and its monoexponential fitting: y = 0.233×exp(–*t*/167), where S_i is the area beneath the TA band, 460–550 nm (*b*). λ_{exc} = 430 nm, T = 295 K.

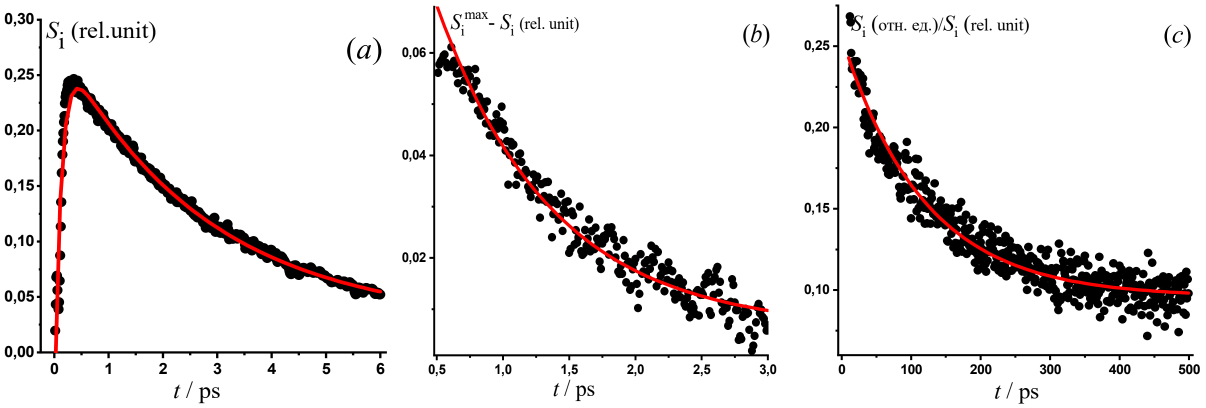

Figure S2. TA band (**CD6**·$(Sr^{2+})_2$ in MeCN) accumulation at 416 nm, time plot and its biexponential fitting: y = –0.344×exp(–*t*/0.124)+0.26×exp(–*t*/2.71)+0.026, where S_i is the area beneath the TA band, 400–450 nm (*a*). TA band (**CD6**·$(Sr^{2+})_2$ in MeCN) accumulation at 500 nm, time plot and its monoexponential fitting: y = 0.112×exp(–*t*/0.87)+0.006, where S_i is the area beneath the TA band, 500–650 nm (*b*). TA band (**CD6**·$(Sr^{2+})_2$ in MeCN) decay at 500 nm, time plot and its monoexponential fitting: y = 0.156×exp(–*t*/125)+0.095, where S_i is the area beneath the TA band, 400–650 nm (*c*). λ_{exc} = 340 nm, T = 295 K.

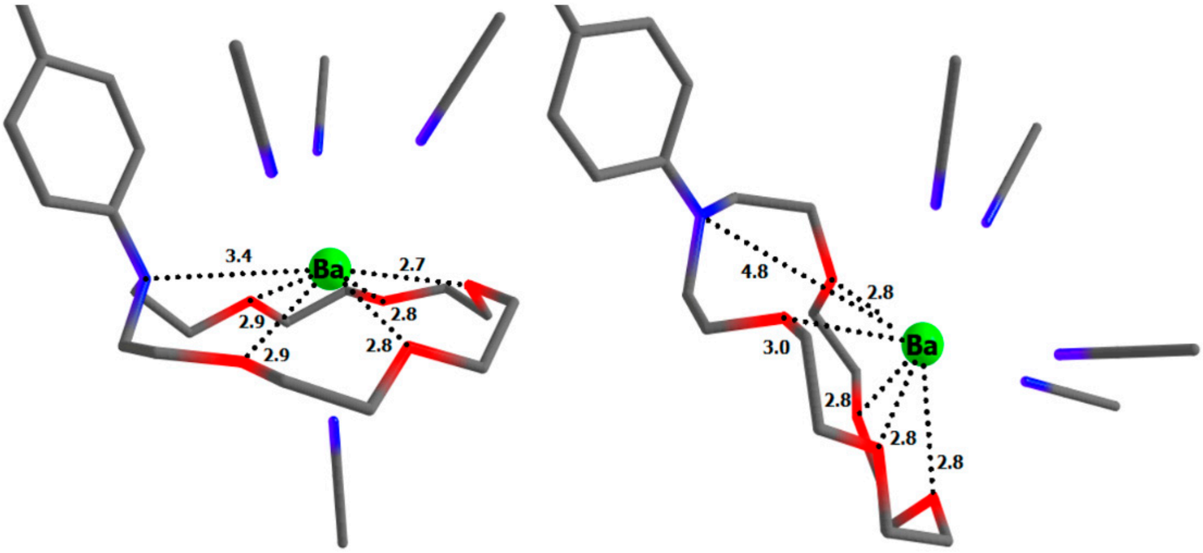

Figure S3. Partial **CD6**·$(Ba^{2+})_2$ structure: prior to photoinduced recoordination (axial conformation, (3+1) MeCN solvation shell) (left); after photoinduced recoordination (equatorial conformation, (4+0) MeCN solvation shell) (right); Ba^{2+} (green), N (blue), O (red), C (grey) H atoms are omitted for clarity.
